# Nanotechnology Approaches for Mitigating Biologic Immunogenicity: A Literature Review

**DOI:** 10.3390/pharmaceutics17070888

**Published:** 2025-07-07

**Authors:** Jouri Alanazi, Fadilah Sfouq Aleanizy, Fulwah Yahya Alqahtani

**Affiliations:** 1 Drug Sector, Saudi FDA, Riyadh 13513, Saudi Arabia; jsanazi@sfda.gov.sa; 2 Department of Pharmaceutics, College of Pharmacy, King Saud University, Riyadh 11495, Saudi Arabia

**Keywords:** anti-drug antibodies (ADAs), monoclonal antibodies (mAbs), zwitterionic poly(carboxybetaine) nanocages, synthetic vaccine particles (SVPs), tolerogenic nanoparticles, lipid nanoparticles (LNPs)

## Abstract

Biologic therapeutics, particularly monoclonal antibodies (mAbs), have revolutionized disease treatment paradigms; however, their clinical success is often hindered by immunogenicity. Host immune recognition of these biologics can induce anti-drug antibody (ADA) formation, leading to reduced therapeutic efficacy, altered pharmacokinetics and serious adverse events, such as infusion reactions and loss of response. Overcoming these immunogenicity challenges is essential to maximize the clinical effect of biologics and ensure patient safety. This paper offers an overview of the mechanisms underlying the formation of anti-drug antibodies and explores potential nanotechnology-based strategies to reduce or eliminate these responses. Specifically, the review examines how the immune system recognizes biologics and develops ADAs, which can impact drug efficacy and safety. The review then investigates various nanotechnology approaches aimed at mitigating ADA formation, potentially improving the therapeutic outcomes of biologic drugs.

## 1. Introduction

Immunogenicity is a significant concern associated with biologic therapies, particularly monoclonal antibodies (mAbs). The host immune system can recognize these biologics as foreign, triggering the development of anti-drug antibodies (ADAs) [1]. This phenomenon is distinctive for biologic drugs and is not observed with small-molecule medications. ADAs can interact with the therapeutic monoclonal antibody (mAb), potentially modifying its pharmacokinetics and biodistribution by increasing its clearance from the body, which may result in lower concentrations of the mAb in the bloodstream or tissues [1]. Consequently, this can diminish the therapeutic effectiveness of the mAb and may lead to hypersensitivity reactions and adverse effects in patients, including anaphylaxis. ADAs have been detected in numerous mAb therapeutics, leading to the termination of clinical trials and the rejection or withdrawal of drugs following approval [1]. Even mAbs derived from human sequences can elicit ADA responses, leading to clinical trial failures [1]. The risk of immunogenicity has been a major obstacle limiting the clinical use of murine mAbs, especially in cancer therapy, where large doses and repeated administrations are often required [2]. Therefore, addressing immunogenicity is critical for maximizing the clinical benefit of mAbs and ensuring patient safety.

Nanotechnology has emerged as a promising approach to address this challenge. Nanomaterials possess unique physicochemical properties that can be leveraged to modulate the immune response [3]. Recent advancements in nanoparticle technology have created new opportunities to decrease the incidence of anti-drug antibody responses to therapeutic proteins [1,4]. Nanoparticles, which are self-assembling entities composed of biodegradable polymers, are identified by the immune system as particles comparable in size to viruses [1,4]. They are transported to lymphoid organs from peripheral tissues by resident antigen-presenting cells (APCs) or are filtered from the bloodstream by the liver and spleen [1,4]. This literature review examines the current state of research on using nanotechnology to overcome biologic immunogenicity, focusing on the underlying mechanisms and key considerations for clinical translation.

## 2. Anti-Drug Antibodies (ADAs): Mechanisms and Clinical Implications

The therapeutic use of antibodies originated in the late 19th century when Emil von Behring established serum treatment for infectious illnesses, despite its associated hazards, including serum sickness and hypersensitivity responses [5,6,7]. The introduction of hybridoma technology by Köhler and Milstein in 1975 facilitated the large-scale manufacture of monoclonal antibodies (mAbs), resulting in the FDA’s approval of the first therapeutic mAb, Orthoclone OKT3, in 1986 [8,9]. This invention initiated an age of monoclonal-antibody-based therapeutics for many clinical applications [8]. To mitigate the substantial immunogenicity associated with murine-derived antibodies, which presents as human anti-murine antibody (HAMA) responses, successive iterations of chimeric, humanized and human antibodies were engineered [6,10]. Monoclonal antibodies (mAbs) are now essential for several therapeutic regimens and are used in the treatment of a wide range of disorders. The conditions include rheumatoid arthritis, Crohn’s disease, hemophilia types A and B, different malignancies, psoriasis and viral disorders, among others [10]. In 2021, the FDA authorized the 100th monoclonal antibody (mAb) therapeutic, and in recent years, mAb therapies have represented 50% of new biologic approvals by the FDA [11,12]. The therapeutic antibody market was valued at roughly USD 238 billion in 2023, with estimations suggesting it may reach around USD 680 billion by 2033 [13].

Despite these advancements, the total eradication of immunogenicity continues to be unattainable. Fully human antibodies have shown the ability to elicit anti-drug antibody (ADA) responses, highlighting the intricacies of host immune surveillance. Chimerization was developed as a technique to reduce the immunogenicity linked to fully murine antibodies [6]. This process entails replacing the constant regions of antibodies with sequences derived from human immunoglobulins while only preserving the variable regions sourced from mice [8]. Ultimately, it became possible to substitute the variable framework regions with human sequences, retaining only the complementarity-determining regions (CDRs) that function as the binding domains of the antibody, which remained of murine origin [8]. This approach notably reduced the immunogenicity of monoclonal antibodies (mAbs); however, residual murine sequences still resulted in the formation of anti-drug antibodies (ADAs) against the chimeric antibodies.

The subsequent advancement included the comprehensive substitution of the antibody gene with human sequences, a process known as humanization. A range of methods were utilized to achieve this goal, focusing mainly on the removal of non-self-sequences identified through a comparative analysis of murine antibodies and their human counterparts [4,14]. Considering the significant function of complementarity-determining regions (CDRs) in antigen binding and antibody functionality, it is crucial to verify that any substituted sequences do not undermine antigen binding affinity [8]. Each amino acid that varies between murine and human antibodies is carefully evaluated for its potential effect on binding affinity if modified [15]. This evaluation can be conducted using in vitro techniques like phage display and yeast display, which assess the impact of amino acid modifications on binding affinity, alongside in silico methods that offer 3D modeling and structural analysis of proposed mutations [14].

CDR grafting specifically involves the replacement of only the CDR regions of antibodies, whereas the modification of both CDR and framework regions, while retaining only the specificity-determining residues (SDRs), is referred to as SDR grafting [8]. A method known as resurfacing concentrates exclusively on replacing residues situated on the surface of the antibody once its secondary structure has been formed [8]. Furthermore, fully human antibodies can be produced using methods such as transgenic mice engineered to express solely human immunoglobulin genes or through libraries obtained from fully synthetic or human donor B cells [6]. The launch of two innovative transgenic mouse models in the 1990s, specifically the HuMAb Mouse and the XenoMouse, represented important progress in the generation of fully human antibodies, despite the subsequent emergence of various other transgenic mouse platforms [16]. Both humanized and fully human antibodies exhibit similar tendencies to induce anti-drug antibodies (ADAs), with some fully human antibodies also not effectively addressing the issue of immunogenicity [6].

## 3. ADAs: Definition and Development

**Figure 1:**
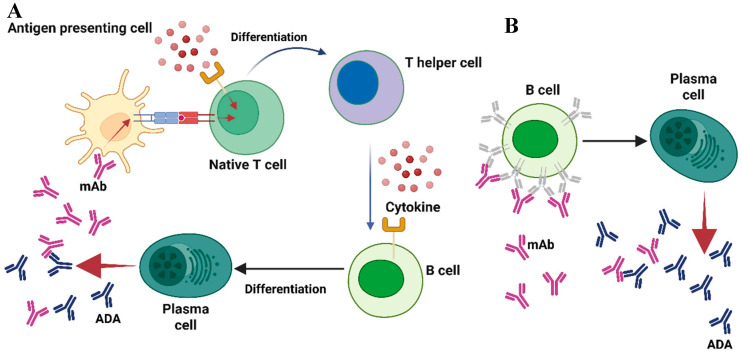
A summary of the stages involved in the formation of ADAs. (**A**) In the T-cell-dependent pathway, antigen-presenting cells (APCs) endocytose the therapeutic monoclonal antibody (mAb) and present linear epitopes within the context of MHC class II to naïve CD4+ T cells. Antigen-presenting cells (APCs) secrete cytokines during the process of antigen presentation, which, in conjunction with the interaction between major histocompatibility complex (MHC) and T-cell receptor (TCR), facilitates the differentiation of naïve T cells into CD4+ T helper cells. Activated T helper cells release cytokines that stimulate B cells to differentiate into plasma cells, initiating the production of antibodies against the therapeutic agent. (**B**) In the T-cell-independent pathway, therapeutic monoclonal antibodies with multiple epitopes directly crosslink B-cell receptors, thereby stimulating differentiation into plasma cells that produce drug-specific antibodies. Adapted from [13] with some modification, generated by BioRender.

Anti-drug antibodies (ADAs) can develop against therapeutic monoclonal antibodies (mAbs) via T-cell-dependent or T-cell-independent mechanisms (Figure 1A,B). In the T-cell-dependent pathway, antigen-presenting cells (APCs), such as dendritic cells (DCs), internalize therapeutic monoclonal antibodies (mAbs) and present linear epitopes alongside MHC class II molecules to naïve CD4+ T cells [17]. During antigen presentation, antigen-presenting cells (APCs) secrete cytokines that, along with the interaction between major histocompatibility complex (MHC) and the T-cell receptor (TCR), promote the differentiation of naïve T cells into CD4+ T helper cells [17,18]. Activated T helper cells release cytokines that facilitate the differentiation of B cells into plasma cells, thereby initiating the production of antibodies against the therapeutic monoclonal antibody [17,18]. The peptides displayed by MHC class II to T cells are known as T-cell epitopes (TCEs). Antibodies produced via T-cell-dependent pathways are predominantly of the IgG isotype, recognized for their durability and high-affinity properties [18]. A subset of B cells that generate anti-drug antibodies will differentiate into memory B cells, which persist over time and can initiate a rapid response to the same antigen upon re-exposure [17].

In cases of T-cell-independent B-cell activation, therapeutic monoclonal antibodies (mAbs) with multiple epitopes can efficiently crosslink B-cell receptors (BCRs), facilitating the release of drug-specific antibodies [18]. This activation mechanism, however, does not produce the strong signals required for isotype switching, leading to the predominance of IgM antibodies. These antibodies are characterized by a shorter half-life and lower affinity for antigens, attributable to the lack of the affinity maturation process [18]. Like T-cell engagers (TCEs), B cells recognize specific antigenic regions referred to as B-cell epitopes (BCEs). These epitopes are generally located on the surface of therapeutic proteins and are primarily conformational [12,17]. The presence of both TCEs and BCEs in a therapeutic mAb may affect the formation of anti-drug antibodies (ADAs). However, the immunogenicity of mAbs can differ considerably among patients and across various disease contexts, suggesting that the existence of epitopes alone does not fully account for immunogenicity [19]. Various factors related to the medication, the patient undergoing treatment and the prescribed dosage regimen may contribute to the emergence of an anti-drug response [13]. Understanding these immune pathways is essential for developing effective strategies to reduce immunogenicity in biologic therapeutics.

## 4. Nanotechnology-Based Strategies for Overcoming Biologic Immunogenicity

Recent advances in nanotechnology have unlocked new possibilities for modulating immune responses to biologic therapies. By leveraging the unique physicochemical properties of nanomaterials, researchers are developing novel strategies to either suppress or reprogram immune recognition of therapeutic proteins. This section critically examines key nanotechnology-based approaches aimed at mitigating biologics’ immunogenicity.

### 4.1. Modulating Immune Responses with Nanomaterials

Nanomaterials may interact with the immune system in complicated manners, resulting in either immunostimulatory or immunosuppressive consequences [20]. Inorganic nanoparticles, including gold and silica, can stimulate immune cells and improve antigen presentation, possibly augmenting the immunological response to a biologic [20]. Conversely, polymer-based nanoparticles may be designed to encapsulate and administer immunosuppressive agents, attenuating the immune response and minimizing the possibility of immunogenicity [20].

### 4.2. PEGylation

The conjugation of bulky hydrophilic materials to proteins is a commonly utilized approach to reducing the immunogenicity of protein drugs by physically shielding their immunogenic epitopes. Initially, hydrophilic polymers such as polyethylene glycol (PEG) were considered immunologically inert and increased circulation time [21]. However, their association with proteins has been demonstrated to introduce an additional factor in immune response through the haptenic effect, resulting in anti-polymer activity. The immune responses to protein-associated polymers result from both the intrinsic immunogenicity of the polymers and their low grafting density on protein surfaces, constrained by the availability of conjugation sites [21]. An increasing number of clinical reports indicate that anti-polymer antibodies, rather than anti-protein antibodies, are often the primary cause of clinical issues in certain cases [22]. In the standard scenario involving Pegloticase (Krystexxa^®^), a PEGylated uricase product, over 40% of patients with refractory chronic gout (RCG) experienced significant anti-polymer antibody responses, resulting in non-responsiveness to the treatment [23]. Furthermore, anti-polymer antibodies diminished the effectiveness of other polymer-conjugated proteins available, such as PEG-asparaginase (Oncaspar^®^) [24,25] and PEG-interferon alfa (Pegasys^®^) [26].

### 4.3. Zwitterionic Poly(carboxybetaine) Nanocages

Zwitterionic materials possess a pair of oppositely charged ions within the same moiety, resulting in an overall neutral charge. These materials are recognized for their significant hydration effects, which are facilitated by the electrostatic interactions between zwitterions and adjacent water molecules [27]. Among zwitterionic materials, poly(carboxybetaine) (PCB), a biomimetic polymer derived from glycine betaine, emerges as a promising super-hydrophilic material for biomedical applications due to its excellent biocompatibility and inertness to biological systems [27,28,29]. Recently, PCB was utilized to enhance the stability and immunological properties of proteins [21,30,31,32]. The results indicated the absence of anti-PCB antibodies following intravenous administrations of a PCB-modified protein, whereas a significantly elevated level of anti-PEG antibodies was observed in groups injected with the PEGylated counterpart, suggesting that PCB is a material with minimal immunogenicity [21,30,31,32].

A recent study [30] presents a novel PCB-based inverse nanoemulsion [33] method for the physical encapsulation of proteins within nano-sized zwitterionic networks (PCB NC), which are polymerized using CB-derived monomers [21] and a bifunctional crosslinker [34]. This method offers a more generalized and straightforward approach to protein modification compared to earlier techniques that rely on chemical reactions with the protein surface. It allows for the non-invasive protection of proteins, independent of surface conjugation, while maintaining their structural integrity [30]. In their study, to demonstrate that PCB NC could eradicate immune responses, they conducted repetitive intravenous injections of native uricase, PEGylated uricase (PEG-uricase) and PCB-NC-encapsulating uricase (PCB-uricase) in healthy Sprague Dawley (SD) rats over five consecutive weeks (one dose per week). Rat sera were collected on the 35th day for antibody tests [30]. The results of their study showed that uricase encapsulated in PCB NC effectively eliminated the anti-protein or anti-polymer (anti-PCB) antibody responses, as no detectable IgM or IgG (<1:200) against either uricase or PCB was observed following five administrations. A substantial anti-protein (anti-uricase) antibody response was observed in the native uricase group, with IgM titers at 1:6400 and IgG titers exceeding 1:25,600. PEGylation reduced anti-protein IgM and IgG titers to 1:400 and 1:3200, respectively, while simultaneously inducing the production of anti-polymer (anti-PEG) antibodies, with IgM titers at 1:6400 and IgG titers at 1:1600 [30]. This observation aligns with clinical findings associated with Pegloticase. Anti-polymer antibodies, rather than anti-protein antibodies, are responsible for the observed loss of efficacy of Pegloticase in clinical settings [32].

The percentage of activated APC in the PCB-uricase group was comparable to that in the unstimulated control group [30]. The absence of stimulation from APC resulted in a significant reduction in IL-4 levels in the PCB-uricase group. Meanwhile, in the groups treated with uricase and PEG-uricase, a significant increase in the ratio of stimulated APC and an increase in the secretion of IL-4, indicative of stimulated T-cell proliferation, were observed.

These results, consistent with findings from Ab tests, indicate that PCB NCs may inhibit APC activation and consequently attenuate the immune response cascade. This effect is likely achieved through the reduction in protein endocytosis, interference with protein proteolysis and physical blockage of MHCII-epitope binding. Gouty rats administered native uricase and PEG-uricase exhibited a limited degree of disease improvement. In comparison, rehabilitation in the PCB-uricase group was significantly accelerated, evidenced by a rapid decline in clinical scores [30]. The fifth injection of PCB-uricase significantly reduced the level of pro-inflammatory cytokines as well as the leukocyte number in synovial fluids, confirming that PCB-uricase could still efficiently catalyze the metabolism of urate and lessen the deposition of inflammation incentive MSU at knee joints even after five repeated administrations. Moreover, histopathological analysis of periarticular tissues indicates a significant efficacy of PCB-uricase in alleviating gouty arthritis, as evidenced by a marked reduction in the proliferation of lining cells around the synovium and minimal cartilage loss observed [30]. The cohorts receiving native uricase and PEG-uricase demonstrated a significantly elevated histologic score and more severe osteoarthritis (OA), marked by moderate multifocal destruction of the femoral cartilage. A systemic evaluation of pharmacokinetic and pharmacodynamic profiles reveals that both native uricase and PEGylated uricase had a significant decline in circulation time and urate-eliminating capacity following multiple administrations. This decline indicates a loss of efficacy and correlates with increased antibody titers. In contrast, PCB-uricase exhibited a remarkable circulation duration and improved pharmacodynamic effect following five consecutive intravenous injections. The findings align with the PK results, indicating that serum levels of PCB-uricase are significantly higher than those of native uricase and PEG-uricase, attributable to its slower elimination rate and extended residence time in the bloodstream [30]. The prolonged presence of PCB NC in systemic circulation, as demonstrated by pharmacokinetic and biodistribution studies, supports the sustained pharmacodynamic effect of PCB-uricase in facilitating urate metabolism [30]. Moreover, PCB-uricase demonstrates enhanced distribution in other primary organs, particularly the liver and spleen. The non-fouling property of PCB NC partially accounts for the ability of PCB-uricase to evade rapid clearance by the reticuloendothelial system, resulting in prolonged and elevated accumulation in the liver and spleen [30]. Their study represents the first confirmed strategy to effectively mitigate the efficacy loss associated with existing polymer-conjugated biologics. This is significant for clinical application due to the increasing prevalence of anti-protein and anti-polymer antibodies [30].

### 4.4. Synthetic Vaccine Particles (SVPs)

The formation of anti-drug antibodies (ADAs) frequently leads to treatment failure and adverse events, including hypersensitivity reactions, related to biologic therapies. Consequently, it is essential to prevent ADAs in a manner that is specific to the antigen to enhance the safety and efficacy of marketed products. In the study by Ferrari et al. (2015) [35], the application of SVPs—polymeric, synthetic, biodegradable nanoparticles that deliver the tolerogenic immunomodulator rapamycin to promote lasting, antigen-specific immune tolerance—was studied. In mice, the co-injection of SVPs with free antigen via intravenous or subcutaneous routes leads to significant CD4+ T-cell and B-cell tolerance, characterized by the suppression of their activation across multiple challenges, an elevation in regulatory cells and the prevention of antigen-specific hypersensitivity reactions [35]. Only encapsulated rapamycin, as opposed to its free form, can induce immunological tolerance to various antigens. Co-injections of SVP and antigen in rats and cynomolgus monkeys also induce B-cell tolerance. In mice that spontaneously develop rheumatoid arthritis (RA), treatment with SVP and adalimumab prevents the formation of anti-adalimumab antibodies, thereby normalizing adalimumab pharmacokinetics and enhancing both clinical and histological manifestations of RA [35]. SVP therapy constitutes an innovative antigen-specific strategy aimed at preventing anti-drug antibodies (ADAs) in response to biologic therapies, in addition to addressing allergies and autoimmune disorders [35].

### 4.5. Tolerogenic Nanoparticles (ImmTOR)

**Figure 2:**
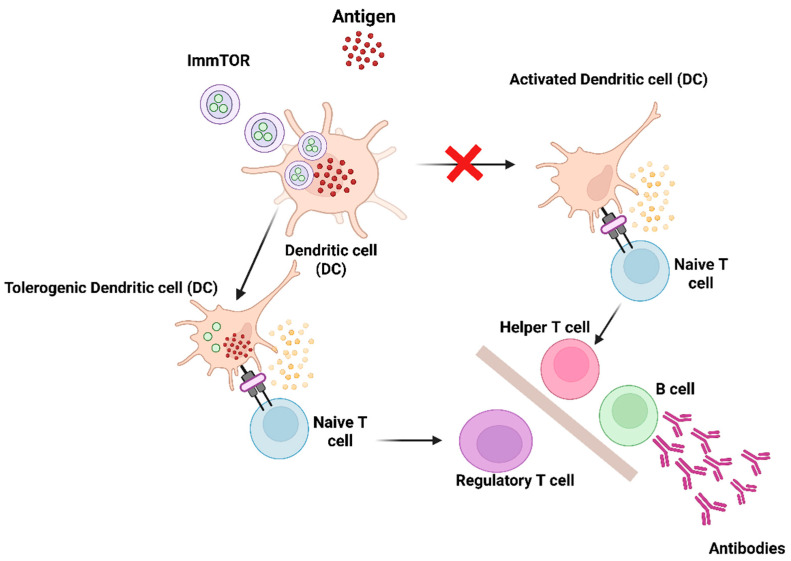
Diagram illustrating the mechanism of action of ImmTOR. ImmTOR is specifically internalized by antigen-presenting cells, including dendritic cells (DCs) located in the spleen and liver. ImmTOR promotes the generation of tolerogenic dendritic cells that process and present co-administered antigens, leading to the expansion of antigen-specific regulatory T cells. Tregs suppress the activation of effector T cells and inhibit the development of anti-drug antibodies. Adapted from [13] with some modification, generated by BioRender.

Pegylated uricases, including pegloticase and pegadricase, are enzyme therapies that efficiently metabolize uric acid and represent promising treatment options for adult patients with chronic gout who are resistant to conventional therapies [36,37,38,39,40]. The primary therapeutic objective in gout management is to lower serum uric acid levels (sUA) to below 6 mg/dL. Elevated uric acid levels can lead to the crystallization of monosodium urate (MSU) crystals in joints and soft tissues, resulting in gout flares, bone remodeling and significant pain [41]. The current oral therapies are insufficient for the effective elimination of large MSU crystal deposits, known as tophi, since serum uric acid levels must be sustained significantly below 6 mg/dL to facilitate the dissolution of urate crystals [40]. Pegylated uricase, in contrast, can lower serum uric acid levels to below 2 mg/dL, leading to a more rapid resolution of tissue monosodium urate deposits [39]. Nonetheless, pegylated uricases exhibit significant immunogenicity, leading to the development of anti-drug antibodies (ADAs) in approximately 90% of patients [23,36], associated with diminished therapeutic efficacy and heightened infusion reactions [36]. In this regard, biodegradable nanoparticles encapsulating rapamycin (ImmTOR), an mTOR pathway inhibitor, have been developed to induce selective immune tolerance to co-administered biologic drugs [1,42]. ImmTOR, formerly referred to as synthetic vaccine particle (SVP)-rapamycin, consists of synthetic, biodegradable nanoparticles made from PLA (poly(D,L-lactide)) and PLA-PEG (poly(D,L-lactide)-block-poly(ethylene-glycol)) polymers that encapsulate rapamycin. Rapamycin inhibits the activation of effector T cells and is utilized clinically in chronic immunosuppressive regimens for preventing renal allograft rejection [43]. In vitro studies indicate that rapamycin can induce tolerogenic antigen-presenting cells (APCs), which facilitate the activation of regulatory T cells [44]. ImmTOR nanoparticles exhibit selective biodistribution to the spleen and liver after intravenous administration, where they are internalized by antigen-presenting cells (APCs) [42,45,46,47]. ImmTOR induces a tolerogenic phenotype in dendritic cells within the spleen and in liver sinusoidal endothelial cells, as well as other antigen-presenting cells in the liver. The co-administration of ImmTOR with antigen leads to the induction or expansion of antigen-specific regulatory T cells (Tregs) (Figure 2) [42,46,48]. The induction of specific immune tolerance by ImmTOR is substantiated by the following evidence: (1) ImmTOR promotes the induction of Tregs that are specific to the co-administered antigen [42,46,48]; (2) tolerance can be transferred through the adoptive transfer of splenocytes from treated animals to naïve recipients [47,48,49]; (3) this tolerance persists even after subsequent exposure to the antigen alone [42,46,47]; and (4) animals that have been tolerized to a specific antigen retain the ability to respond to an unrelated antigen [42,46,47,49]. Preclinical studies demonstrated that ImmTOR can reduce the immunogenicity of pegadricase, leading to sustained control of sUA in uricase-deficient mice [42]. A multi-center, double-blind, placebo-controlled study was conducted to evaluate the safety, tolerability, ADA formation and pharmacodynamic effects of a combination of fixed-dose pegadricase (0.4 mg/kg) with escalating doses of ImmTOR (0.03–0.3 mg/kg) [50].

The results revealed that the co-administration of ImmTOR with pegadricase led to a dose-dependent reduction in anti-uricase ADAs [50]. Patients administered 0.15 and 0.3 mg/kg doses of ImmTOR sustained serum uric acid levels below the therapeutic threshold of 6 mg/dL for a minimum duration of 30 days [50]. This was associated with sustained serum pegadricase activity and reduced ADA titers. ImmTOR inhibited the formation of anti-PEG antibodies, which were observed to be transient and less frequent in treated patients. Patients maintaining low serum uric acid (sUA) levels gradually returned to baseline by day 51, without a resurgence of anti-drug antibodies (ADAs), indicating enzyme clearance rather than immune escape [50]. ImmTOR presents a novel, feasible approach to prevent ADA formation, enabling sustained therapeutic activity and potentially allowing monthly dosing regimens [50]. Beyond gout, ImmTOR’s platform holds promise for mitigating immunogenicity across a broad range of biologic treatments, including monoclonal antibodies, enzyme replacement therapies, recombinant immunotoxins and gene therapies [50].

### 4.6. Lipid-Nanoparticle-Encapsulated mRNA-Encoding Monoclonal Antibodies

The endogenous expression of therapeutic proteins implies the production of these proteins within the patient’s body, using the patient’s cellular machinery. One method for endogenously expressing a monoclonal antibody involves the utilization of a viral vector or nucleic acids, such as messenger RNA (mRNA) and DNA [51]. It is hypothesized that in vivo production of the mAb will result in the incorporation of post-translational processing that is endogenous to the patient, rather than patterns derived from exogenous producer cell lines [51].

**Figure 3:**
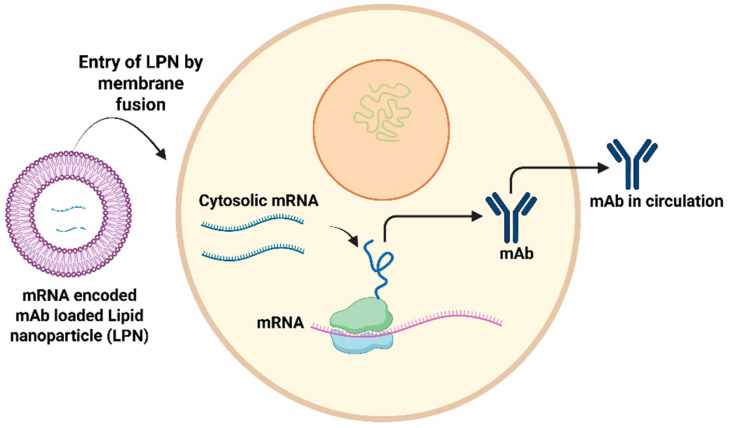
Summary of the mechanism through which monoclonal antibodies (mAbs) are synthesized within host cells following the introduction of RNA-encoded mAbs via lipid nanoparticle. Adapted from [13] with some modification, generated by BioRender.

Multiple preclinical studies have reported mRNA lipid nanoparticle (LNP) antibody delivery (Figure 3), and more recently, safety and pharmacokinetics have been evaluated in a phase 1 human trial. Research demonstrates that IVT-synthesized mRNA encoding the therapeutic tau antibody, RNJ1, facilitates endogenous translation, resulting in the production of functional full-length therapeutic antibodies upon delivery to human neuroblastoma cells [52]. mRNA-encoded monoclonal antibodies encapsulated in lipid nanoparticles demonstrate prolonged expression levels. For instance, mRNA antibodies targeting orthopoxviruses, encapsulated in lipid nanoparticles and administered intravenously to mice, demonstrated efficacy [53]. The current poxvirus therapeutics indicate that antibody therapies for orthopoxviruses significantly enhance survival rates by mitigating weight loss and tissue viral load, effectively protecting mice from severe disease [53]. The mRNA–antibody approach demonstrated simplicity, cost effectiveness and the provision of immediate protection, alongside flexibility [53,54]. An mRNA lipid nanoparticle containing an anti-Chikungunya virus monoclonal antibody (mRNA-1944) was assessed in a phase 1 clinical trial, with no instances of anti-drug antibody development reported [55].

## 5. Considerations for Clinical Translation

### 5.1. Biocompatibility and Safety

Biocompatibility and safety are essential for clinical translation. Lipid nanoparticles (LNPs) typically exhibit favorable safety profiles; however, there are ongoing concerns regarding rare hypersensitivity reactions and long-term biodistribution [56,57]. Zwitterionic coatings exhibit low immunogenicity in animal studies; however, extensive human safety data are required [27,29].

### 5.2. Scale-Up and Manufacturing

Manufacturing that is both scalable and reproducible is crucial for clinical translation. Advancements in microfluidic production have enhanced the large-scale manufacturing of LNPs, ensuring consistency [58]. The optimization of zwitterionic polymer synthesis and conjugation to proteins is essential for industrial scalability [27,29].

### 5.3. Regulatory Challenges

The regulatory framework for nanomedicine continues to change. Agencies necessitate a comprehensive characterization of nanomaterials, consistency across batches and substantial safety and efficacy data. The immunogenicity of both the nanomaterial and the therapeutic payload requires thorough evaluation, as emphasized by recent FDA guidance on nanotechnology products [59].

## 6. Conclusions

Biologic therapies have revolutionized the management of numerous diseases; however, immunogenicity remains a significant challenge to their long-term efficacy and safety. The development of anti-drug antibodies (ADAs) not only compromises therapeutic outcomes but also increases the risk of adverse events, underscoring an urgent need for innovative mitigation strategies. Nanotechnology offers a promising avenue for mitigating these challenges. Emerging approaches, including zwitterionic poly(carboxybetaine) nanocages, tolerogenic nanoparticles (NPs), synthetic vaccine particles and nanoparticles encapsulating mRNA-encoding monoclonal antibody, demonstrate significant potential in attenuating immune recognition of biologic therapeutics. Nanotechnology can mitigate biologics from immune recognition. These strategies have shown promising preclinical and early clinical results, indicating their capacity to enhance drug stability, prolong pharmacokinetics and reduce immunogenicity without broadly suppressing immune function. The key factors for the effective translation of these nanotechnologies encompass ensuring biocompatibility and safety, creating scalable and reproducible manufacturing processes and addressing changing regulatory frameworks. The intricacy of immune responses to the therapeutic payload and the nanomaterial requires thorough preclinical and clinical assessment. A comprehensive understanding of the relationship between nanomaterial properties, immune modulation and clinical outcomes is crucial for maximizing the potential of these strategies in addressing the immunogenicity of biologic drugs and enhancing patient outcomes. Further research is needed to fully realize the potential of nanotechnology in this area and translate these strategies into clinical applications.
